# ﻿*Artemisiacalcicola* (Asteraceae, Anthemideae), a new species from karst region in Guizhou, southwestern China

**DOI:** 10.3897/phytokeys.213.96203

**Published:** 2022-11-16

**Authors:** Cheng-Sheng Li, Xiao-Rui Chi, Xin-Qiang Guo, Long Wang

**Affiliations:** 1 Key Laboratory of Plant Resources Conservation and Sustainable Utilization, South China Botanical Garden, Chinese Academy of Sciences, Guangzhou 510650, China; 2 University of Chinese Academy of Sciences, Beijing 100049, China; 3 College of Life and Environmental Sciences, Hangzhou Normal University, Hangzhou 311121, China; 4 Zhejiang Provincial Key Laboratory for Genetic Improvement and Quality Control of Medicinal Plants, Hangzhou Normal University, Hangzhou 311121, China

**Keywords:** Compositae, limestone flora, morphology, taxonomy

## Abstract

*Artemisiacalcicola* (Asteraceae, Anthemideae), a new species from karst region in Shibing county, Guizhou province, southwestern China, is described and illustrated. The species can be readily assigned to A.subg.Artemisia in having fertile disk florets and glabrous receptacles. Within this subgenus, *A.calcicola* is distinguished by having (2- or) 3-pinnatipartite leaves and narrowly ellipsoid involucres 0.9–1.3 mm in diameter. It resembles *A.annua* to some extent, but differs immediately by the plant duration, stem and leaf indumentum, and involucre shape and size. A detailed description and distribution map of this species are also provided herein.

## ﻿Introduction

*Artemisia* L. (Asteraceae), the largest genus of the tribe Anthemideae, comprises 300–500 species mainly distributed in the northern hemisphere (Ling 1991; Shulz 2006; Ling et al. 2011; Pellicer et al. 2014, 2018; Malik et al. 2017). China is considered one of the most important species centers of this genus, with ca. 190 species and 40 varieties recorded (Ling 1988, 1991; Ling et al. 2011; Shultz and Boufford 2012; Guo et al. 2020, 2021, 2022). This genus is well known for containing various remarkable bioactive compounds, especially the efficient antimalarial agent artemisinin extracted from the leaves of *A.annua* L. (Tu 2011, 2017; Pellicer et al. 2018).

**Figure 1.**
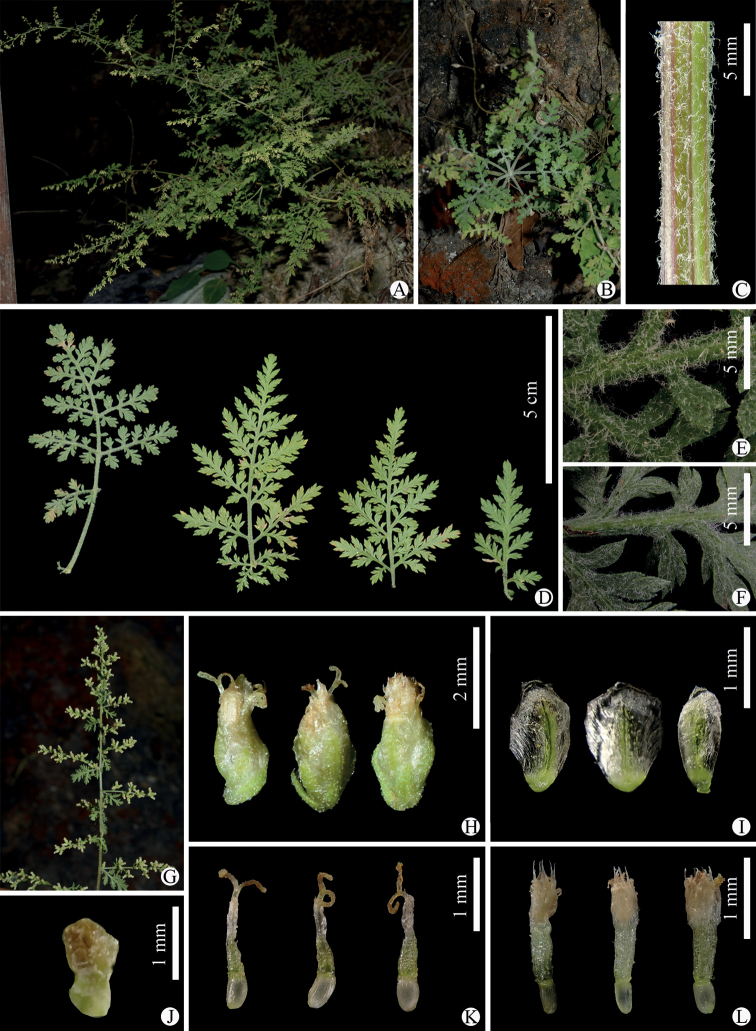
*Artemisiacalcicola* sp. nov. **A** habitat and habit **B** leaf rosette of a vegetative branch **C** portion of stem **D** leaves **E** adaxial side of leaf **F** abaxial side of leaf **G** portion of synflorescence **H** capitula **I** phyllaries (abaxial side) **J** receptacle **K** marginal female florets **L** disk florets. All photographs by Long Wang.

During a botanical trip to Guizhou in southwestern China in 2021, we discovered an unusual population of *Artemisia* in a karst region in Shibing (Fig. 1). At first glance, the plants were easily referred to A.subg.Artemisia due to their fertile disk florets and glabrous receptacles. Further critical observations revealed that they are rather distinct within this subgenus by having (2- or) 3-pinnatipartite leaves and narrowly ellipsoid involucres 0.9–1.3 mm in diameter. Morphologically, they are superficially similar to *A.annua*, a species in the same subgenus and widely distributed in the northern hemisphere, in having (2- or) 3-pinnatipartite stem leaves, ovate-acuminate or ovate, entire or (1- or) 2-toothed leaf lobules, and a narrow to broad panicle-like synflorescence (Fig. 1), but differ markedly by being perennial (vs. annual) and by having arachnoid-tomentose (vs. glabrous or sparsely pubescent) stems and leaves, narrowly ellipsoid (vs. globose or hemispheric) involucres 0.9–1.3 mm (vs. 1.5–2.5 mm) in diameter (Table 1). We therefore determined that the population in question represents a hitherto undescribed species, which we name *A.calcicola* and describe below.

**Table 1.** Morphological comparison between *Artemisiaannua* and *A.calcicola* sp. nov.

	*A.annua*	*A.calcicola*
Duration	Annual	perennial
Stem	glabrous, sparsely pubescent	arachnoid-tomentose
Leaf	glabrous, sparsely pubescent; middle stem leaves 3 (or 4)-pinnatipartite; segments 5–8 (−10) pairs	arachnoid-tomentose; middle stem leaves (2- or) 3-pinnatipartite; segments 3–6 pairs
Capitulum	shortly pedunculate	sessile or subsessile
Involucre	globose to hemispheric; 1.5–2.5 mm in diameter	narrowly ellipsoid; 0.9–1.3 mm in diameter
Marginal female floret	10–20	4–6
Disk floret	10–30; corolla yellow or dark yellow	7–9; corolla creamy yellow

## ﻿Materials and methods

For morphological comparison, we critically examined physical or digitalized herbarium specimens of the genus *Artemisia* deposited at several major herbaria in China including CDBI, HNWP, IBSC, KUN, NAS, PE, SZ, and WUK (acronyms follow Thiers (2022)). Plants of *A.calcicola* were collected and photographed during our 2021 field investigation to Guizhou province. Morphological observations and measurements were based on fresh materials as well as herbarium specimens deposited at IBSC.

## ﻿Taxonomic treatment

### ﻿ Artemisia calcicola

Taxon classificationPlantaeAsteralesAsteraceae

X.Q.Guo & L.Wang sp. nov.

47EE5355-CA6A-5A07-B465-D2DB84C45AE5

urn:lsid:ipni.org:names:77308334-1

Figs 1 , 2

#### Diagnosis.

*Artemisiacalcicola* is distinguished within the A.subg.Artemisia in having (2- or) 3-pinnatipartite leaves and narrowly ellipsoid involucres 0.9–1.3 mm in diameter. Within this subgenus, it is merely superficially similar to *A.annua* in having (2- or) 3-pinnatipartite stem leaves, ovate-acuminate or ovate, entire or (1- or) 2-toothed leaf lobules, and a narrow to broad panicle-like synflorescence, but differs by being perennial and by having arachnoid-tomentose stems and leaves and narrowly ellipsoid involucres 0.9–1.3 mm in diameter (a detailed morphological comparison between the two species is given in Table 1).

#### Type.

**Figure 2.**
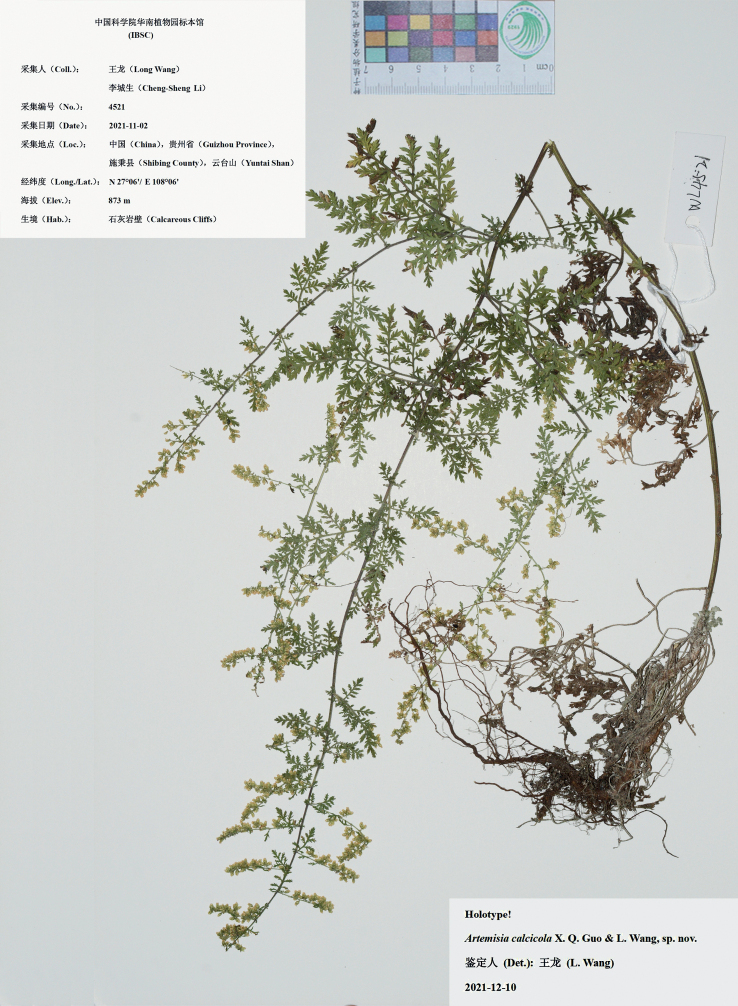
Holotype sheet of *Artemisiacalcicola* sp. nov.

China. Guizhou: Shibing, Yuntai Shan, 27°06'N, 108°06'E, calcareous cliffs, 873 m a.s.l., 12 October 2021 (fl.), *Long Wang & Cheng-Sheng Li 4521* (holotype: IBSC; isotypes: IBSC, PE). Fig. 2.

#### Description.

Herbs, perennial, 40–80 (–100) cm tall. Rhizome woody, up to 0.7 cm in diameter at base. Stems arachnoid-tomentose, more or less branched, erect or ascending. Basal stem leaves usually withered at anthesis, petiolate; (2- or) 3-pinnatipartite. Middle stem leaves petiolate; petiole 1–3 cm long; leaf blade ovate or ovate-oblong, 3–7 cm long, 3–5 cm broad, light green adaxially, grayish green abaxially, arachnoid-tomentose on both surfaces, (2- or) 3-pinnatipartite; segments 3–6 pairs, elliptic or ovate-elliptic, 1–2.5 cm long, 0.5–1 cm broad; lobes 3–6 pairs on each segment, elliptic or ovate, 5–8 mm long, 3–5 mm broad, with lobules ovate-acuminate or ovate, entire or (1- or) 2-toothed. Upper stem leaves subsessile or sessile; leaf blade ovate or ovate-elliptic, 3–5 cm long, 3–4 cm broad, (2- or) 3-pinnatipartite, arachnoid-tomentose on both sides; segments 3–7 pairs, elliptic or ovate, 1–2 cm long, 0.5–1.5 cm broad; lobes 3–5 pairs on each segment, elliptic or ovate, 0.5–1 cm long, 3–5 mm broad, with lobules ovate-acuminate or ovate, entire or (1- or) 2-toothed. Uppermost stem leaves subsessile; leaf blade ovate or ovate-elliptic, 1.5–2 cm long, 1.5–2 cm broad, (2- or) 3-pinnatipartite, arachnoid-tomentose on both sides; segments 3–6 pairs, elliptic or ovate, 0.5–1 cm long, 0.5–1 cm broad; lobes 2–4 pairs on each segment, ovate, with lobules ovate-acuminate or ovate, apex mucronate, entire or (1- or) 2-toothed. Synflorescence a narrow or broad panicle. Capitula sessile or subsessile, usually 3–7 clustered together. Involucres narrowly ellipsoid, 1.8–2.2 mm high, 0.9–1.3 mm in diameter. Phyllaries 3–4 rows, abaxially sparsely arachnoid-pubescent (outermost row) to glabrous (inner rows), obovate, ovate-oblong to elliptic, green when fresh, margin membranous. Receptacle glabrous. Marginal female florets 4–6, ca. 2 mm long, fertile; corolla tubular, 0.7–1 mm long, apex 2-toothed; style exserted. Disk florets 7–9, ca. 2 mm long, bisexual, fertile; corolla creamy yellow, 0.8–1 mm long, apex 5-toothed. Achenes cylindrical. Pappus absent.

#### Distribution and habitat.

**Figure 3.**
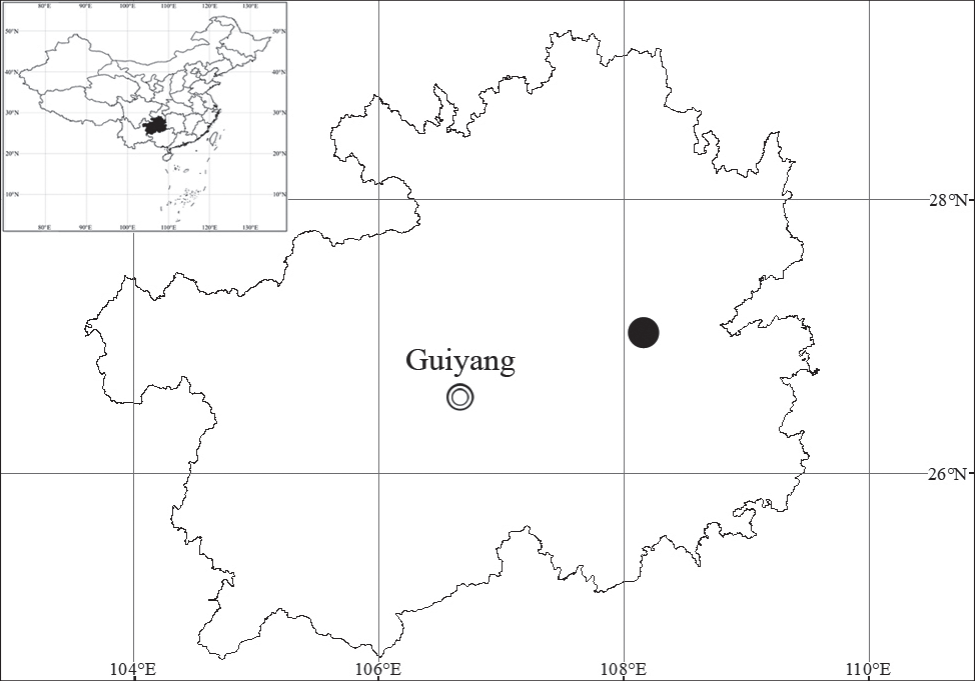
Distribution of *Artemisiacalcicola* sp. nov. (black circle).

*Artemisiacalcicola* is currently known only from the type locality, i.e. Yuntai Shan in Shibing, Guizhou, southwestern China (Fig. 3). It grows on calcareous cliffs at an altitude of ca. 900 m above sea level.

#### Etymology.

Latin *calcis*, genitive singular of *calx*, limestone, and *cola*, *dweller*, alluding to habitat on calcareous cliffs.

#### Phenology.

Flowering from October to November; fruiting from November to December.

#### Vernacular name.

灰岩蒿 (Chinese pinyin: huī yán hāo).

#### Conservation status.

*Artemisiacalcicola* is currently known only from its type locality, i.e. Yuntai Shan in Shibing, Guizhou, southwestern China. The single population we discovered consists of no more than 20 individuals. Before acquiring adequate information to make a conclusive assessment of its risk of endangerment, the conservation status of *A.calcicola* is here recommended as “Data Deficient (DD)” (IUCN 2019).

#### Notes.

In Artemisiasubg.Artemisia, *A.calcicola* is also somewhat similar to *A.lancea* Vaniot, a species widely distributed in China, India, Japan, Korea, and Russia, particularly in the narrowly ellipsoid involucres. However, *A.calcicola* differs remarkably from *A.lancea* by an array of characters, including the arachnoid-tomentose (vs. glabrescent or sparsely arachnoid) stems, (2- or) 3-pinnatipartite (vs. 1-pinnatisect to 3-partite, or undivided), arachnoid-tomentose (vs. adaxially sparsely arachnoid, abaxially densely tomentose) stem leaves, and elliptic or ovate (linear-lanceolate or linear if divided) leaf segments.

## Supplementary Material

XML Treatment for Artemisia calcicola

## References

[B1] Guo XQ Wang L Yang QE (2020) Taxonomic notes on *Artemisiawaltonii* (Asteraceae, Anthemideae), with reduction of *A.kangmarensis* and *A.conaensis* to the synonymy of its type variety. Phytotaxa 450(2): 149–172. 10.11646/phytotaxa.450.2.2

[B2] Guo XQ Wang L Yang QE (2021) *Artemisiaflaccida* (Asteraceae, Anthemideae) is merged with *A.fulgens*, with transfer of A.flaccidavar.meiguensis to *A.fulgens*. Phytotaxa 514(3): 221–237. 10.11646/phytotaxa.514.3.3

[B3] Guo XQ Wang L Yang QE (2022) Clarification of morphological characters and geographical distribution of *Artemisianeosinensis* (Asteraceae, Anthemideae), a strikingly misunderstood species from China. Phytotaxa 544(1): 11–36. 10.11646/phytotaxa.544.1.2

[B4] IUCN [Standards and Petitions Subcommittee] (2019) Guidelines for Using the IUCN Red List Categories and Criteria. Version 15.1. Prepared by the Standards and Petitions Subcommittee. [https://nc.iucnredlist.org/redlist/content/attachment_files/RedListGuidelines.pdf]

[B5] Ling YR (1988) The Chinese *Artemisia* Linn.–– the classification, distribution and application of *Artemisia* Linn. in China. Bulletin of Botanical Research 8(4): 1–61.

[B6] Ling YR (1991) *Artemisia* L. In: Ling Y Ling YR (Eds) Flora Reipublicae Popularis Sinicae.Vol. 76 (2). Science Press, Beijing, 1–253.

[B7] Ling YR Humphries CJ Gilbert MG (2011) *Artemisia* L. In: Wu ZY Raven PH Hong DY (Eds) Flora of China.Vol. 20–21. Science Press, Beijing & Missouri Botanical Garden Press, St. Louis, 676–737.

[B8] Malik S Vitales D Hayat MQ Korobkov AA Garnatje T Vallès J (2017) Phylogeny and biogeography of ArtemisiasubgenusSeriphidium (Asteraceae: Anthemideae). Taxon 66(4): 934–952. 10.12705/664.8

[B9] Pellicer J Hidalgo O Garnatje T Kondo K Vallès J (2014) Life cycle versus systematic placement: Phylogenetic and cytogenetic studies in annual *Artemisia* (Asteraceae, Anthemideae). Turkish Journal of Botany 38: 1112–1122. 10.3906/bot-1404-102

[B10] Pellicer J Saslis-Lagoudakis CH Carrió E Ernst M Garnatje T Grace OM Gras A Mumbrú M Vallès J Vitales D Rønsted M (2018) A phylogenetic road map to antimalarial *Artemisia* species. Journal of Ethnopharmacology 225: 1–9. 10.1016/j.jep.2018.06.030 29936053

[B11] Shultz LM Boufford DE (2012) A new species of *Artemisia* (Asteraceae: Anthemideae) from Sichuan, China. Harvard Papers in Botany 17(1): 21–23. 10.3100/025.017.0106

[B12] Shulz LM (2006) *Artemisia* L. Flora of North America. Vols. 19–21. Oxford University Press: New York, 503–534.

[B13] Thiers B (2022) Index Herbariorum: A global directory of public herbaria and associated Staff. http://sweetgum.nybg.org/science/ih/ [accessed 10 August 2022]

[B14] Tu YY (2011) The discovery of artemisinin (qinghaosu) and gifts from Chinese medicine. Nature Medicine 17(10): 1217–1220. 10.1038/nm.2471 21989013

[B15] Tu YY (2017) From *Artemisiaannua* L. to artemisinins. Chemical Industry Press, Academic Press, London, 426 pp. 10.1016/B978-0-12-811655-5.00027-1

